# Protective Effect of Selenium-Based Medicines on Toxicity of Three Common Organophosphorus Compounds in Human Erythrocytes *In Vitro*

**DOI:** 10.22074/cellj.2016.3846

**Published:** 2016-01-17

**Authors:** Maryam Sadat Fakhri-Bafghi, Seyedeh Farnaz Ghasemi-Niri, Sara Mostafalou, Mona Navaei-Nigjeh, Maryam Baeeri, Azadeh Mohammadirad, Mohammad Abdollahi

**Affiliations:** 1 School of Medicine, Tehran University of Medical Sciences, Tehran, Iran; 2 Faculty of Pharmacy and Pharmaceutical Sciences Research Center, Tehran University of Medical Sciences, Tehran, Iran; 3 Department of Tissue Engineering, School of Advanced Technologies in Medicine, Tehran University of Medical Sciences, Tehran, Iran

**Keywords:** Human Erythrocyte, Organophosphorus, Oxidative Stress

## Abstract

**Objective:**

Organophosphorus (OP) compounds are used to control pests, however they can reach the food chain and enter the human body causing serious health problems by means of acetylcholinesterase (AChE) inhibition and oxidative stress (OS). Among the OPs, chlorpyrifos (CHP), malathion (MAL), and diazinon (DIA) are commonly used for commercial extermination purposes, in addition to veterinary practices, domestic, agricul- ture and public health applications. Two new recently registered medicines that contain selenium and other antioxidants, IMOD and angipars (ANG), have shown beneficial ef- fects for OS related disorders. This study examines the effect of selenium-based medi- cines on toxicity of three common OP compounds in erythrocytes.

**Materials and Methods:**

In the present experimental study, we determined the ef- ficacy of IMOD and ANG on OS induced by three mentioned OP pesticides in human erythrocytes *in vitro*. After dose-response studies, AChE, lipid peroxidation (LPO), total antioxidant power (TAP) and total thiol molecules (TTM) were measured in eryth- rocytes after exposure to OPs alone and in combined treatment with IMOD or ANG.

**Results:**

AChE activity, TAP and TTM reduced in erythrocytes exposed to CHP, MAL and DIA while they were restored in the presence of ANG and IMOD. ANG and IMOD reduced the OPs-induced elevation of LPO.

**Conclusion:**

The present study shows the positive effects of IMOD and ANG in re- duction of OS and restoration of AChE inhibition induced by CHP, MAL and DIA in erythrocytes *in vitro*.

## Introduction

Organophosphorus (OP) compounds are frequently used worldwide. These compounds have shown harmful effects on humans and other living organisms. Widespread use of OPs causes serious worldwide concern since they can enter the food chain and living systems, resulting in several health problems. Other than hazards of acute exposure to OPs, these compounds are able to affect human health through chronic exposure. The belief is that chronic exposure to OPs may result in human hormonal disorders and debilitating disorders such as diabetes, infertility, cancer, and neurodegenerative diseases (1). OPs are believed to generate oxidative stress (OS) and inhibit the enzyme acetylcholinesterase (AChE) (2). OS may compromise many vital functions and induce cell injuries, all of which ultimately result in serious problems. Many human diseases are thought to be related to OS (3). 

Chlorpyrifos (CHP), malathion (MAL), and diazinon (DIA) are three most commonly used OPs in the environment for agricultural and domestic purposes. 

The mature erythrocyte contains high concentrations of hemoglobin and functions as an oxygen transporter. It has a particular membrane structure, active energy sources within the cell, and an adequate supply of specific phosphorylated intermediates. The erythrocyte retains a collection of enzymes, proteins, carbohydrates, lipid, anions and cations, all of which are required in balance, for effective metabolism of cells. Erythrocytes are known to be very susceptible to OS induced by high oxygen strains, which can result in degenerative changes in hemoglobin, the cell membrane, and enzymes required for normal erythrocyte function (4). OS may block the ionic pump leading to disturbances of ions and water movement from the erythrocyte membrane, which may induce cellular swelling and probable lysis. Furthermore, OS affects the physiology of the erythrocyte and leads to the development of inflammation and related toxicities (5). 

Oxidative damage to an erythrocyte membrane is usually detected by measuring lipid peroxidation (LPO), membrane protein cross-linking, or the combination of both. In order to reduce the harmful effects of OS, it is logical to look for compounds capable of fighting against reactive oxygen species (ROS). Two new selenium-based medicines, IMOD and angipars (ANG), which have been registered and released into the market during the previous 6 years show promising effects on OS-related disorders such as acquired immune deficiency syndrome (AIDS), diabetes, aging, and polycystic ovary syndrome (PCOS) (6,8). 

IMOD, an immunomodulator, is derived from extracts of the *Rosa canina* fruit, *Tanacetum vulgare* leaves, and Utrica dioica leaves enriched with selenium, carotene, flavonoids, and urea, that has been exposed to a pulsed electromagnetic field (9). ANG is comprised of* Melilotus officinalis* extract along with 7-hydroxycoumarin, flavonoids, oleanene glucuronide, urea, selenium and fructose. It contains anti-inflammatory, anti-edematous, and antioxidants properties (10).

We aimed to examine the possible protective effects of these two selenium-enriched drugs against OP-induced oxidative damage to human erythrocytes. 

## Materials and Methods

### Chemicals

Acetylthiocholine iodide, 5,5’-dithiobis-2-nitrobenzoic acid (DTNB) were obtained from Merck (Germany), trichloroacetic acid (TCA), tris base, 1,1,3,3-tetramethoxypropane (MDA), 2-thiobarbituric acid (TBA), 2,4,6-tripyridyl-s-triazine (TPTZ), n-butanol, acetic acid, FeCl3-6H2O, benzethonium chloride (Hyamine® 1622), and phosphate buffer were acquired from Sigma-Aldrich (Germany). Analytical grade forms of CHP, DIA and MAL were obtained from local pesticide manufacturing companies. IMOD and ANG were supplied by Rose-Pharmed (Iran). The enzymelinked immunosorbent assay (ELISA) kits were purchased from Bender MedSystem (Germany). 

### Erythrocyte preparation

This experimental study was approved by the Institute Review Board of Tehran University of Medical Sciences with code number of 90-04-15116052. Fresh human venous blood was obtained from healthy non-smoker volunteers on no current medications. The blood was combined with citrate as an anticoagulant. A total of 5 ml of blood was centrifuged at 2380 g for 10 minutes at 4˚C. The erythrocytes were separated and washed twice with 0.9% NaCl and once with phosphate-buffered saline (PBS, Sigma-Aldrich, Germany), pH=7.4. The supernatant and the buffy coat were carefully removed after each wash. Erythrocytes at 10% hematocrit (10 volumes of erythrocytes to 90 volumes of PBS) were prepared and used for different incubation periods. Incubated erythrocytes in buffer were used as the non-treated control cells. 

### Determination of the half maximal inhibitory concentration (IC_50_)

In order to verify the half maximal IC_50_ of OPs, amounts of 0.5, 1, and 1.5 times the previously measured IC_50_ (11) were prepared. We incubated various concentrations of each OP with the blood samples. The AChE inhibition assay was performed and we determined the IC_50_ values for CHP, DIA and MAL according to Probit Analysis (StatsDirect 3.0.117). The new IC_50_ values were used for subsequent analyses.

### Treatment

Blood samples were prepared and erythrocytes were separated for the next steps. Prior to incubation, we added specific doses (CHP: 9.8 μM, MAL: 71.2 μM, DIA: 24.45 μM) of each OP compound and either IMOD or ANG to the blood samples. The groups included the following: control; IC_50_ concentration of pesticides (CHP or MAL or DIA); IC_50_ concentration of pesticides (CHP or MAL or DIA) with IMOD (10 ppm); and IC_50_ concentration of pesticides (CHP or MAL or DIA) with ANG (10 ppm). After a 4-hour incubation at 37˚C, the mixtures were stored at -20˚C and thawed one day later. After thawing, erythrocytes were damaged by osmotic pressure, and centrifuged. The supernatants were used to determine the mentioned biochemical parameters.

### Acetylcholinesterase activity

AChE activity for the erythrocytes was measured according to the method of George and Abernethy (12) set up previously (13) using benzethonium chloride (Hyamine® 1622). Briefly, we added 10 μl of the sample to 3 ml of solution that contained 25 mM DTNB in 75 mM phosphate buffer. Then, 10 μl of 3 mM acetylcholine iodide was added and absorbance changes were measured at 412 nm in a two-fold ray spectrophotometer. 

### Total antioxidant power (TAP)

The ferric reducing antioxidant power (FRAP) test measures a sample’s ability to reduce Fe^3+^ to Fe^2+^ where the result is considered to be the TAP of the sample. Of the above-mentioned preserved supernatants, we added 0.1 ml of the supernatants to a reaction mixture that consisted of acetate buffer (300 mM, pH=3.6), TPTZ (10 ml in 40 mM HCl), ferric chloride (20 mM) in the proportion of 10:1:1, after which the mixtures were incubated for 10 minutes at 37˚C, and the absorbance measured at 593 nm as described previously (14). The results were expressed as μmol/mg protein.

### Lipid peroxidation

MDA is the final product of the oxidation of polyunsaturated fatty acids (PUFA). It can react with TBA to produce a complex that can be distinguished by spectrometric analysis. We have assessed LPO in the samples in terms of thiobarbituric acid reactive substances (TBARS). Samples were diluted with buffered saline (1:5) and a 400 μl aliquot was mixed with 800 μl of 28% TCA (w/v), then centrifuged at 3000×g for 30 minutes. Next, 600 μl of supernatant was added to 150 μl of TBA (1% w/v). This blend was incubated for 15 minutes in a boiling water bath, after which 4 ml of n-butanol was added; the solution was subsequently centrifuged and cooled as previously described (15). The absorption of the supernatant was measured at 532 nm. Data were expressed as nM. 

### Statistical analysis

Each analysis was repeated a total of four times. Data are presented as mean ± SEM. One-way ANOVA and Tukey’s multi-comparison trials were performed by Stats-Direct 3.0.117 in order to determine the statistical differences. The degree of significance was P<0.05. 

## Results

**Fig.1:**
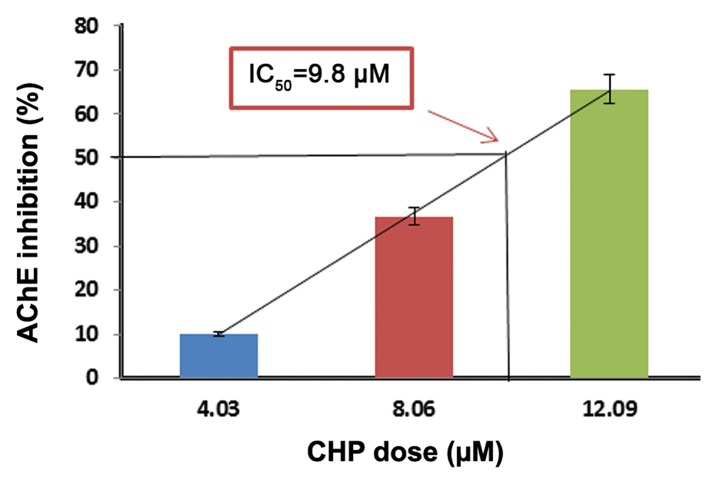
Determination of CHP IC_50_ based on AChE inhibition. Data are expressed as mean ± SEM of three different experiments (each experiment was performed in duplicate). The AChE inhibition by 50% as the IC_50_ of CHP was 9.8 μM. CHP; Chlorpyrifos, IC_50_; Inhibitory concentration 50% and AChE; Acetylcholinesterase.

**Fig.2:**
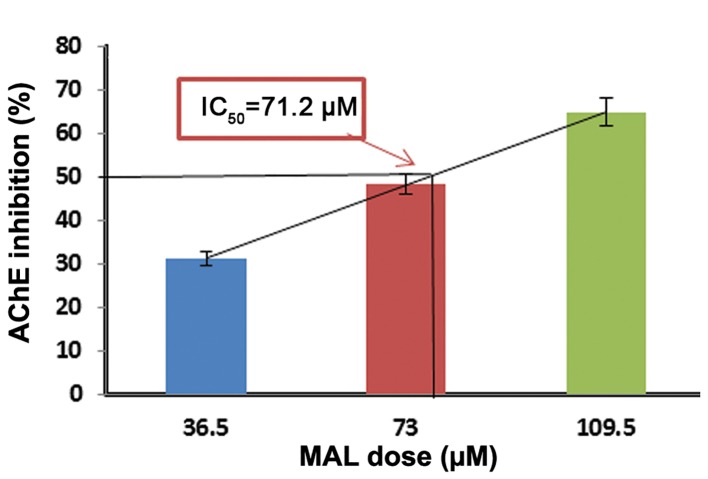
Determination of MAL IC_50_ based on AChE inhibition. Data are expressed as mean ± SEM of three different experiments (each experiment was performed in duplicate). The AChE inhibition by 50% as the IC_50_ of MAL was 71.2 μM. MAL; Malathion, IC_50_; Inhibitory concentration 50% and AChE; Acetylcholinesterase.

**Fig.3:**
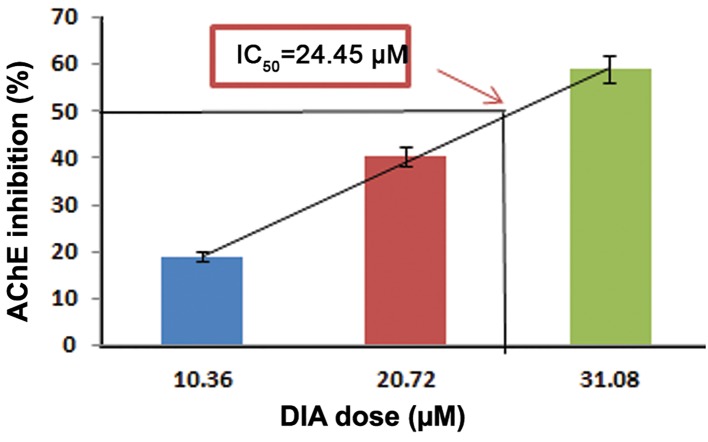
Determination of the DIA IC_50_ based on AChE inhibition. Data are expressed as the mean ± SEM of three different experiments (each experiment was performed in duplicate). The AChE inhibition by 50% as the IC_50_ of DIA was 24.45 μM. DIA; Diazinon, IC_50_; Inhibitory concentration 50% and AChE; Acetylcholinesterase.

We measured the IC_50_values in order to ascertain the effectiveness of these compounds in inhibiting biological or biochemical function by 50%. The values of IC_50_ were 9.8 μM for CHP, 25.45 μM for MAL, and 71.2 µM for DIA (Fig .1-3). 

### Acetylcholinesterase activity

**Fig.4:**
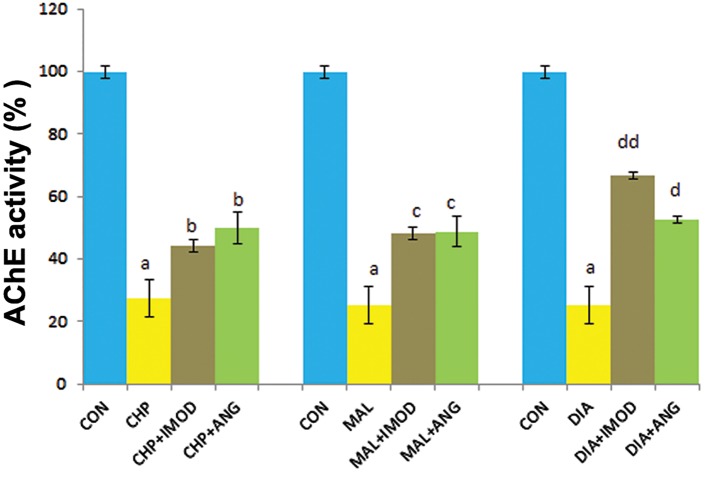
Effect of IMOD and ANG on AChE activity of erythrocytes. Effect of IMOD and ANG on AChE activity of erythrocytes. IMOD and ANG significantly reduced AChE activity. The values are expressed as mean ± SD for the following groups: control (CON); IC_50_ concentration of pesticides (CHP, MAL or DIA); IC_50_ concentration of pesticides (CHP, MAL or DIA) with IMOD (10 ppm); and IC_50_ concentration of pesticides (CHP, MAL or DIA) with ANG (10 ppm). a; Significantly different from CON at P<0.05, b; Significantly different from CHP at P<0.05, c; Significantly different from MAL at P<0.05, d; Significantly different from DIA at P<0.05, dd; Significantly different from DIA at P<0.01, ANG; Angipars, AChE; Acetylcholinesterase, IC_50_; Inhibitory concentration 50%, CHP; Chlorpyrifos, MAL; Malathion and DIA; Diazinon.

Next, we evaluated IMOD and ANG by measurement of AChE activity. AChE activity reduced in erythrocytes in the CHP (P=0.001), MAL (P=0.000), and DIA (P=0.000) groups compared to the control group. IMOD restored the activity of AChE which had been suppressed by CHP (P=0.021), MAL (P=0.0034), and DIA (P=0.003). ANG significantly retrieved AChE activity compared to CHP (P=0.049), MAL (P=0.041), and DIA (P=0.014, Fig .4). 

### Total antioxidant power as ferric reducing antioxidant power

**Fig.5:**
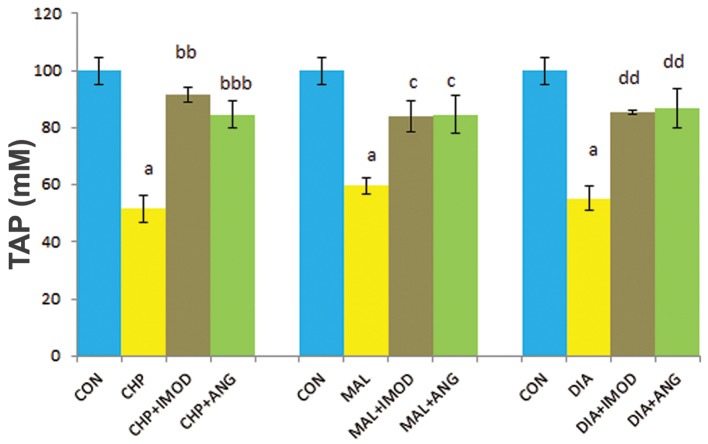
Effect of IMOD and ANG on TAP of erythrocytes. The values are expressed as mean ± SD for the following groups: control (CON); IC_50_ concentration of pesticides (CHP, MAL or DIA); IC_50_ concentration of pesticides (CHP, MAL or DIA) with IMOD (10 ppm); and IC_50_ concentration of pesticides (CHP, MAL or DIA) with ANG (10 ppm). a; Significantly different from CON at P<0.05, bb; Significantly different from CHP at P<0.01, bbb; Significantly different from CHP at P<0.001, c; Significantly different from MAL at P<0.05, dd; Significantly different from DIA at P<0.01, ANG; Angipars, TAP; Total antioxidant power, IC_50_; Inhibitory concentration 50%, CHP; Chlorpyrifos, MAL; Malathion and DIA; Diazinon.

The CHP, MAL and DIA groups had less TAP compared to the control group. IMOD significantly decreased the antioxidant power which had been elevated by CHP (P=0.000), MAL (P=0.040), and DIA (P=0.005). ANG was able to significantly retrieve antioxidant power which had been suppressed by CHP (P=0.02), MAL (P=0.03), and DIA (P=0.007, Fig .5). 

### Lipid peroxidation

**Fig.6:**
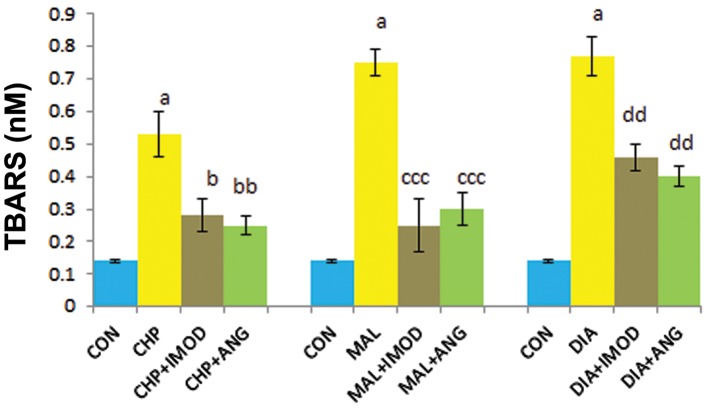
Effect of IMOD and ANG on LPO of erythrocytes. The values are expressed as mean ± SD for the following groups: control (CON); IC_50_concentration of pesticides (CHP, MAL or DIA); IC_50_concentration of pesticides (CHP, MAL or DIA) with IMOD (10 ppm); and IC_50_concentration of pesticides (CHP, MAL or DIA) with ANG (10 ppm). a; Significantly different from CON at P<0.05, b; Significantly different from CHP at P<0.05, bb; Significantly different from CHP at P<0.01, ccc; Significantly different from MAL at P<0.001, dd; Significantly different from DIA at P<0.01, ANG; Angipars, LPO; Lipid peroxidation, IC_50_; Inhibitory concentration 50%, CHP; Chlorpyrifos, MAL; Malathion, DIA; Diazinon and TBARS; Thiobarbituric acid reactive substances.

TBARS, as an indicator of LPO increased in the CHP (P=0.000), MAL (P=0.000), and DIA (P=0.000) groups compared to the control group. IMOD significantly decreased the amount of TBARS which was elevated by CHP (P=0.01), MAL (P=0.000), and DIA (P=0.001) in erythrocytes. ANG significantly retrieved LPO when compared to CHP (P=0.023), MAL (P=0.000), and DIA (P=0.000, Fig .6). 

### Total thiol molecules (TTM)

**Fig.7:**
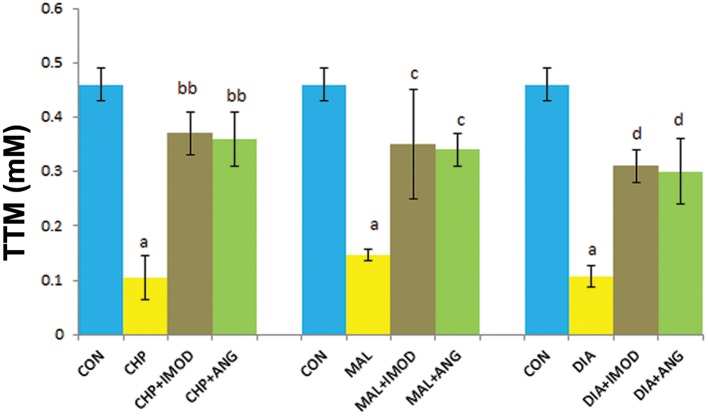
Effect of IMOD and ANG on TTM of erythrocytes. The values are expressed as mean ± SD for the following groups: control (CON); IC_50_concentration of pesticides (CHP, MAL or DIA); IC_50_concentration of pesticides (CHP, MAL or DIA) with IMOD (10 ppm); and IC_50_concentration of pesticides (CHP, MAL or DIA) with ANG (10 ppm). a; Significantly different from CON at P<0.05, bb; Significantly different from CHP at P<0.01, c; Significantly different from MAL at P<0.05, d; Significantly different from DIA at P<0.05, ANG; Angipars, TTM; Total thiol molecules, IC_50_; Inhibitory concentration 50%, CHP; Chlorpyrifos, MAL; Malathion and DIA; Diazinon.

We observed a reduction in TTM in the CHP (P=0.000), MAL (P=0.000), and DIA (P=0.000) groups compared with the control group. IMOD significantly restored TTM that had been reduced by CHP (P=0.004), MAL (P=0.036), and DIA (P=0.014). ANG significantly reduced TTM compared to CHP (P=0.006), MAL (P=0.046), and DIA (P=0.021, Fig .7, Table 1).

**Table 1:** The values represent the mean ± SD of the examined markers of the groups (control, CHP, CHP+IMOD, CHP+ANG, MAL, MAL+IMOD, MAL+ANG, DIA, DIA+IMOD, DIA+ANG) and AChE activity, TAP, LPO, TBARS and TTM


	Control	CHP	CHP+IMOD	CHP+ANG	MAL	MAL+ANG	MAL+ANG	DIA	DIA+IMOD	DIA+ANG

AChE	100±2	27.2±6	44±2	50±5	25.36±6	48.29±2	48.78±5	25.36±6	66.66±1	52.63±1
TAP	99.8±4.8	51.56±4.6	91.62±2.7	84.5±4.8	59.56±2.67	83.81±5.5	84.62±6.7	55.25±4.3	85.56±0.7	86.7±7.01
TBARS	0.14±0.007	0.53±0.07	0.28±0.05	0.25±0.03	0.75±0.04	0.25±0.08	0.3±0.05	0.77±0.06	0.46±0.04	0.4±0.03
TTM	0.46±0.03	0.105±0.04	0.37±0.04	0.36±0.05	0.146±0.01	0.35±0.1	0.34±0.03	0.107±0.02	0.31±0.03	0.3±0.06


CHP; Chlorpyrifos, ANG; Angipars, MAL; Malathion, DIA; Diazinon, AChE; Acetylcholinesterase, TAP; Total antioxidant power, LPO; Lipid peroxidation, TBARS; Thiobarbituric acid reactive substances and TTM; Total thiol molecules.

## Discussion

Recent studies have demonstrated that erythrocytes are fragile and this could be of importance since erythrocytes constitute the majority of cells in the body. Although they are endowed with a high level of antioxidant enzymes and molecules, their membranes are susceptible to damage by many environmental chemicals such as OP pesticides (16). Previously, several studies have shown that OPs increase the production of ROS and affect the activity of antioxidant enzymes that result in oxidative damage. The imbalance between the antioxidant elements and ROS can cause LPO as a main feature of OS (17).

In this work, we measured AChE activity, LPO, and TAP in erythrocytes exposed to three common OPs-CHP,MAL and DIA. Next we evaluated the protective ability of IMOD and ANG in retrieving the mentioned criteria. We have observed that AChE activity in erythrocytes was markedly inhibited by CHP, MAL, and DIA. AChE plays an important role in protecting the integrity of erythrocytes. In the case of paroxysmal nocturnal hemoglobinuria (PNH), the activity of this enzyme is markedly decreased in erythrocytes (18). However, pre-incubation with IMOD and ANG decreased the inhibitory effects of CHP, MAL and DIA on AChE activity in erythrocytes. 

OPs have been reported to induce OS through increased formation of ROS and alteration of membrane integrity. MDA is the major product of peroxidized PUFA and increased MDA content is a valuable indicator of LPO (19). In this study, MDA levels have increased after exposure to OPs, while IMOD and ANG treatment attenuated the increased MDA levels. Strong antioxidant properties have been reported for both IMOD and ANG through increasing TAP and decreasing LPO (20). The IMOD complex is comprised of *Rosa canina, Tanacetum vulgare* and *Urtica dioica*. Strong antioxidant properties of IMOD have inspired researchers to evaluate its beneficial effects in OSand inflammatory-based disorders such as acute sepsis (21), colitis (22), diabetes (23), oral lichen planus (24), PCOS (25), and pancreatic langerhans islet transplants (26). 

*Urtica dioica* has been shown to reduce inflammatory cytokines such as tumor necrosis factor-α (TNF-α) and interleukin-1β (IL-1β) at the transcription level (27). Its benefits have been reported in streptozotocin (STZ)-induced diabetes in mice models by stimulation of insulin secretion (28). *Rosa canina* extract has also been reported to have radical scavenging and anti-inflammatory potentials which can be helpful in different oxidant-related diseases (6). Tanacetum vulgare exerts antioxidant and anti-inflammatory effects that contain flavonoids and phenolic compounds with antiviral activity against herpes simplex viruses 1 and 2 (HSV-1 and HSV-2) (29). Flavonoids such as α-tocopherol have the capability to scavenge some radical species directly or act as chain breaking antioxidants (30). 

One of the main components of IMOD and ANG is selenium which can be coupled with copper and zinc to activate metallo-enzymes such as glutathione peroxidase (GPx), superoxide dismutase (SOD), and catalase (CAT) during the cellular defense process against oxidants and associated elements. Selenocompounds are effective in protecting plasmid DNA from peroxynitrite-induced single-strand breaks (31). 

Another change in the cell response to stress is thiol. We have observed that CHP, MAL and DIA caused decreased thiol level in the erythrocytes which was retrieved by IMOD and ANG. In addition to antiinflammatory and anti-apoptotic effects, IMOD has been shown to increase the thiol content that assists with the production of glutathione, cysteine, and hemocysteine, which have numerous roles in metabolism and constitute the most important antioxidant defenses in mammalians (32). 

ANG contains *Melilotus officialis* extract which has been reported to reduce inflammation, regulate the immune system and improve vascular blood flow (33,34). Plesca-Manea et al. (35) reported that coumarin, the main element of ANG, decreased gingival LPO and had inhibitory effects on the formation of DNA damage and synthesis of nitric oxide (NO) in phagocytes. A number of clinical trials have been performed to confirm the antioxidant effect of *Melilotus officialis* and ANG in humans (7). 

## Conclusion

The present *in vitro* study confirms the ability of IMOD and ANG to ameliorate OPs (CHP, MAL and DIA)-induced human erythrocyte toxicity represented by AChE activity in association with oxidative/anti-oxidative components. 
